# Recurring Translocations in Barrett’s Esophageal Adenocarcinoma

**DOI:** 10.3389/fgene.2021.674741

**Published:** 2021-06-09

**Authors:** Manisha Bajpai, Anshuman Panda, Kristen Birudaraju, James Van Gurp, Amitabh Chak, Kiron M. Das, Parisa Javidian, Hana Aviv

**Affiliations:** ^1^Department of Medicine-Gastroenterology and Hepatology, Rutgers-Robert Wood Johnson Medical School, Rutgers The State University of New Jersey, New Brunswick, NJ, United States; ^2^Rutgers Cancer Institute of New Jersey, New Brunswick, NJ, United States; ^3^Cytogenetics Laboratory, Department of Pathology, Rutgers-Robert Wood Johnson Medical School, Rutgers The State University of New Jersey, New Brunswick, NJ, United States; ^4^Department of Pathology, Rutgers-Robert Wood Johnson Medical School, Rutgers The State University of New Jersey, New Brunswick, NJ, United States; ^5^Division of Gastroenterology and Hepatology, University Hospitals Cleveland Medical Center, Case Western Reserve University School of Medicine, Cleveland, OH, United States

**Keywords:** Barrett’s epithelium, esophageal adenocarcinoma, chromosome translocation, fluorescence *in situ* hybridization, biomarkers

## Abstract

Barrett’s esophagus (BE) is a premalignant metaplasia in patients with chronic gastroesophageal reflux disease (GERD). BE can progress to esophageal adenocarcinoma (EA) with less than 15% 5-year survival. Chromosomal aneuploidy, deletions, and duplication are early events in BE progression to EA, but reliable diagnostic assays to detect chromosomal markers in premalignant stages of EA arising from BE are lacking. Previously, we investigated chromosomal changes in an *in vitro* model of acid and bile exposure-induced Barrett’s epithelial carcinogenesis (BEC). In addition to detecting changes already known to occur in BE and EA, we also reported a novel recurring chromosomal translocation t(10:16) in the BE cells at an earlier time point before they undergo malignant transformation. In this study, we refine the chromosomal event with the help of fluorescence microscopy techniques as a three-way translocation between chromosomes 2, 10, and 16, t(2:10;16) (p22;q22;q22). We also designed an exclusive fluorescent *in situ* hybridization for esophageal adenocarcinoma (FISH-EA) assay that detects these chromosomal breakpoints and fusions. We validate the feasibility of the FISH-EA assay to objectively detect these chromosome events in primary tissues by confirming the presence of one of the fusions in paraffin-embedded formalin-fixed human EA tumors. Clinical validation in a larger cohort of BE progressors and non-progressors will confirm the specificity and sensitivity of the FISH-EA assay in identifying malignant potential in the early stages of EA.

## Introduction

Esophageal adenocarcinoma (EA) is a deadly disease with less than 15% 5-year survival that has increased in incidence worldwide in the last two decades (Brown et al., 2008; Jung, 2011). Barrett’s epithelium (BE) is a precancerous lesion in patients with chronic gastroesophageal reflux and poses a 120-fold higher risk of developing EA (Haggitt, 1994; Lagergren et al., 1999; Wild and Hardie, 2003). BE follows a histological progression from metaplasia → low-grade dysplasia (LGD) → high-grade dysplasia (HGD) → EA. The relative risk of developing adenocarcinoma increases with advanced histological grade. While only 0.2–0.5% of patients with non-dysplastic BE are considered “at risk,” those with LGD face a 13% annual risk and the patients with HGD have a 40% 5-year risk of progression to EA (Reid et al., 1988a; Spechler et al., 2011a,b). Due to lack of reliable biomarkers, detection of EA mostly takes place at an advanced stage when prognosis is extremely poor (Schlansky et al., 2006; Timmer et al., 2013). While the recommendations for BE endoscopic surveillance are well defined (Reid et al., 1988b; Wang and Sampliner, 2008), histologic BE staging suffers from inter- and intraobserver variability (Spechler, 2005). The reliability and adequacy of current methods for EA surveillance is therefore controversial (Spechler, 2020), and reliable “biomarkers” for accurate prediction of risk of progression from BE to EA are necessary (Gorospe and Wong Kee Song, 2016).

Barrett’s epithelium is replete with mutations and chromosomal aberrations (Barrett et al., 1999; Frankell et al., 2019) that facilitate development of neoplasia and progression to EA. Aberrations represented by loss or gain of chromosomes or chromosomal regions, loss of heterozygosity (LOH), microsatellite instability, single nucleotide polymorphisms, and epigenetic changes have been widely investigated in primary BE and EA as biomarkers of disease progression (Barrett et al., 1996; Sanz-Ortega et al., 2003; Prasad et al., 2008; Timmer et al., 2014; Li et al., 2015; Secrier et al., 2016). However, very few promising molecular markers have been clinically validated (Souza and Spechler, 2021) and none are currently approved for clinical use. A biomarker panel combining LOH17p, LOH9p, and ploidy was one of the first to clinically test aggregate chromosomal abnormalities and successfully identify risk for neoplastic progression in BE with 85% specificity and sensitivity (Reid et al., 2001). The panel has limited clinical adaptation because it requires multicolor flow cytometry not found in most clinical laboratories. Subsequently, a multicolored (red, green, gold, and cyan) fluorescence *in situ* hybridization (FISH) assay for esophageal cancer was developed that includes probes for 17q12 (ERBB2), 8q24 (MYC), 9p21 (p16), and 20q13 (ZNF217) (Brankley et al., 2006) and is used in esophageal brushings instead of conventional biopsy samples. This panel has a sensitivity of 50, 82, and 100% in detecting LGD, HGD, and EA with a specificity of 67% in predicting response to endoscopic therapy (Timmer et al., 2014). However, neither assay detects chromosome translocations or gene fusions. Gene fusions that are amendable to therapeutic interventions are significant but rare in solid tumors. Some like the *TMPRSS2-ERG* fusion in prostate cancer (Tomlins et al., 2005), *EML4-ELK* in lung (Soda et al., 2007), breast (Stephens et al., 2009), and several other cancers (Kumar-Sinha et al., 2015) are now known targets for therapy. Our group was first to report chromosome translocations in malignant human BE. We identified the translocation t(10:16) in addition to the other known chromosomal aberrations, loss of Y, dup(11)(q13q25), and trisomy 7, 19, and 20 in an *in vitro* model of gastroesophageal reflux-induced BE carcinogenesis (BEC) model (Bajpai et al., 2012). These chromosomal rearrangements are specific and reliable primary “biomarkers” and may represent novel oncogenic gene fusions that alter gene expression and serve as potential therapeutic targets.

Here, we describe a set of three recurring translocations t(2;10)(p22;q22), t(10;16)(q22;q22), and t(2;16)(p22;q22); development of a fluorescence *in situ* hybridization for esophageal adenocarcinoma (FISH-EA) assay with specific probes that span the translocation breakpoints using cells from the *in vitro* BEC model; and detection of one of the fusions resulting from t(2:16) in primary human esophageal adenocarcinoma tumors.

## Materials and Methods

### Cell Culture

The Barrett’s epithelial carcinogenesis model (BEC) was derived from the BAR-T, a non-neoplastic telomerase-immortalized human Barrett’s epithelial cell line (gift from Dr. Rhonda Souza, UT-Southwestern, Texas) (Jaiswal et al., 2007). The BAR-T cells were exposed to acidified (pH 4) 200 μM, bile salt, glycochenodeoxycholicacid (GCDA) (ABS) for 5 min every day over 60 weeks, as described elsewhere (Das et al., 2011) and were frozen in liquid nitrogen at regular intervals. The BEC20W cells revived from liquid N_2_ were grown for additional 20 weeks with and without further ABS exposure in six replicates and screened every 2 weeks for the presence of translocations.

### Chromosome Preparation

Metaphase chromosomes were prepared from the BAR-T (PD ∼45), BEC20W (PD ∼60), and BEC20W + 14W (PD ∼70) cells using standard protocol (Howe et al., 2014). Briefly, 60% confluent cells were incubated overnight with 10 μl/ml of colcemid, trypsinized and mixed with hypotonic solution (0.075 M KCI) at 37°C for 10 min. The cells were fixed with freshly made Carnoy’s solution (3:1 absolute methanol:glacial acetic acid) several times before dropping the concentrated cell pellet onto precleaned microscope slides. The slides were air dried before hybridization.

### Chromosome Paint

Metaphase cell preparations from BAR-T (PD ∼45), BEC20W (PD ∼60), and BEC20W + 14W (PD ∼70) were hybridized with whole chromosome paint probes for chromosomes 2 (red), 10 (red and green), and 16 (green) obtained from Rainbow Scientific (Windsor, CT). The chromosome paint probes hybridize to whole chromosomes, enabling identification of structural aberrations in metaphase cells due to differences in color (Ried et al., 1998). The cells were counterstained with DAPI (blue dye).

### Bacterial Artificial Chromosome Library Screening

The BAR-T cells (negative for translocations) and BEC60W cells (positive for translocations) were used to screen for the appropriate bacterial artificial chromosome (BAC) clones (derived from RPCI-11 human genomic DNA library) using the chromosome walking method. Overlapping BAC clones traversing the breakpoints on Chr2p22, Chr10q22, and Chr16q22 were identified from the UCSC Genome browser and obtained from Empire Genomics, Buffalo, NY after customized fluorescent labeling with Rhodamine (RED) or Fluorescein (Green).

### Human Esophageal Adenocarcinoma Tissue Procurement and Classification

Deidentified human esophageal adenocarcinoma biopsy specimens were obtained from an IRB-approved tissue repository and retrieval service at The Rutgers Cancer Institute of NJ. The tissues were either formalin fixed, paraffin embedded (FFPE), or snap frozen in liquid nitrogen. All 10 tissues used were described as moderately differentiated (T3, Nx) adenocarcinoma arising from Barrett’s esophagus. Details of prior management or time of sample collection were not available. Most of the tissue was tumor with little traces of normal adjacent tissues as marked by three independent pathologists on the serially stained H&E slides. Three non-dysplastic Barrett’s biopsies with paired normal squamous samples were obtained from Case Western Reserve University and BETRNet. Representative H&E of normal, Barrett’s and EAC are provided as Supplementary Figures 1–3.

### Fluorescence *in situ* Hybridization and Enumeration of Signals

Appropriate FISH probes were identified using metaphase chromosomes of the BAR-T and BEC model cells before testing paraffin-embedded (PE) human EAC tissues. The PE tissues were cut into 4–5 μm sections and mounted on positively charged salinized slides. Adjacent sections were used for pathology (H&E) and FISH. Hybridization with FISH probes was performed per standard manufacturer recommendation; briefly, the probes were suspended in 10 μl of hybridization buffer, placed on the slides, and sealed with coverslips. The slides were placed in the automated HYBrite slide processing system (Vysis) for denaturation (3 min at 83°C) followed by overnight hybridization at 37°C. Slides were counterstained with 10 μl DAPI, and FISH signals were counted using Olympus BX41 fluorescent microscope equipped with dual- or triple-band pass filter for DAPI, Red and Green spectra, and Metasystems software for digital conversion and analysis. FISH signals were scored independently by KB, MB, and HA. In each experiment, breakpoint regions of two chromosomes were labeled in red and green. Two fluorescent signals of the same color were interpreted as “normal signal.” Three or more signals of the same color (red or green) were interpreted as either gain of a chromosome or a translocation without fusion with the other chromosome. A yellow signal formed by superimposition of red and green signals from two different chromosomes was counted as “positive signal” for translocation/fusion. Signals from at least 25 cells were recorded per hybridization. FISH-EA probes were separated into three sets to detect three translocation breakpoints: set 1: (2p22;16q22), set 2: (2p22;10q22), and set 3: (10q22;16q22).

### RNA Sequencing and Focused Profiling of Transcriptional Landscape

Total RNA was isolated from BEC0W, BEC20W, BEC40W, and BEC60W cells using the RNeasy mini kit (Qiagen, Redwood City, California) and checked for quality by a bioanalyzer before and after depletion of ribosomal RNA using RiboMinus kit (Invitrogen, Grand Island, NY). After library preparation (per Illumina instructions), single-end 100-bp sequencing was performed on Illumina HiSeq 2000 to generate FPKM data of 20,941 genes (Supplementary Table 1). Systematic error was eliminated through median adjustment, i.e., the median of all genes was set to 1 in each sample. The data was then log normalized as expression = log10 (1 + 99 ^∗^ median-adjusted FPKM), such that the genes that are not expressed at all in these cell lines have the normalized expression = 0, and the median of all genes that are expressed have a normalized expression = 2, in each sample. This data was used for log10 fold change estimation of genes located on chromosomes 2, 10, and 16.

## Results

### Barrett’s Epithelial Cells Exposed to Acid and Bile Develop Recurring Translocations Before Malignant Transformation

**FIGURE 1:**
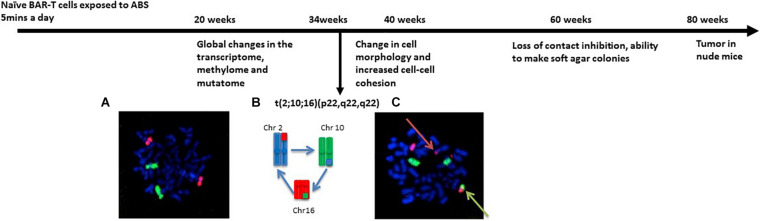
Longitudinal events in Barrett’s epithelial carcinogenesis model. The naïve BAR-T cells exposed to acidified bile salt (ABS) 5 min everyday demonstrate increased columnar phenotype after 2 weeks that remains elevated at the subsequent time points. Although the BEC20W cells are similar to the parent BAR-T cells in their elongated shape and distribution on cell culture dishes, they demonstrate distinct changes in the transcriptome, methylome, and mutatome (Bajpai et al., 2012). With continued ABS exposure, distinct chromosomal changes were observed recurrently in the BEC34-week cells (Bajpai et al., 2013). After 40 weeks of ABS exposure, cells become oval in shape and form distinct clusters on culture dishes. Around 60 weeks, they make soft agar colonies and become malignantly transformed into tumors in nude mice at ∼80 weeks (Das et al., 2011). **(A)** Normal metaphase chromosomes of BEC20-week cells, painted with green (Chr10) and red (Chr16) whole chromosome fluorescent paints. The blue DAPI staining also reveals the normal Chr2 pairs. **(B)** Model of the translocations involving breakage and exchange of chromosome segments between Chr2 (blue), Chr10 (green), and Chr16 (red). **(C)** BEC40W cells with the hybrid red-green chromosome fusion t(10;16) (green arrow) and extra-red signal (red arrow) represent a segment of Chr16 translocated on Chr2 (blue). All metaphase chromosomes are stained blue with DAPI.

Using whole chromosome paints for Chr10 (green) and Chr16 (red), we detected fusion of Chr10 and Chr16 in the BEC40W cells (Figure 1C). Additionally, a red signal from chromosome 16 was detected on the short arm of chromosome 2, showing that the translocation t(10;16) observed by G-banding was not reciprocal but a three-way translocation involving chromosomes 2, 10, and 16 (Figure 1B). Chromosomes 2, 10, and 16 were not translocated in the BAR-T cells growing in parallel without any A + B exposure for 20 weeks (BEC20W) (Figure 1A).

The same translocations recurred in six independent replicates of BEC20W cells exposed to ABS. The t(2;10;16) (p22;q22;q22) appeared as early as 14 weeks after re-exposure to ABS (i.e., BEC20W + 14W), and four of six replicates also had trisomy 20 (Table 1). Additional ABS exposure up to a total of 20 weeks did not add any reproducible changes to the karyotypes of these cells. A parallel set of BEC20W cells growing without further ABS exposure did not show these translocations.

**TABLE 1:** Karyotypes of the six replicates of BEC20W cells exposed to ABS for 14 more weeks.

**Well#**	**BEC20W cells growing for 14 weeks without further A + B exposure**	**BEC20W cells further exposed to A + B for 14 more weeks**
1	47,XY,add(4)(p16.1),add(7)(p22),i(8)(q10),+20	48,XY,i(8)(q10),t(2;10;16)(p22;q22;q22),+20,+20
2	47,XY,add(4)(p16.1),add(7)(p22),i(8)(q10), + 20	47,XY,add(7)(p22),i(8)(q10),+20
3	47,XY,add(4)(p16.1),add(7)(p22),i(8)(q10),+20	48,XY,i(8)(q10),t(2;10;16)(p22;q22;q21), + 20,+20
4	47,XY,add(4)(p16.1),add(7)(p22),i(8)(q10),+20	47,XY,add(7)(p22),i(8)(q10),+20
5	47,XY,add(4)(p16.1),add(7)(p22),i(8)(q10),+20	48,XY,i(8)(q10),t(2;10;16)(p22;q22;q21), + 20, + 20
6	47,XY,add(4)(p16.1),add(7)(p22),i(8)(q10),+20	48,XY,i(8)(q10),t(2;10;16)(p22;q22;q21),+20,+20

### DNA Probes Spanning the Chromosome Breakpoints 2p22, 10q22, and 16q22 Were Identified From Human Bacterial Artificial Chromosome Library

**FIGURE 2:**
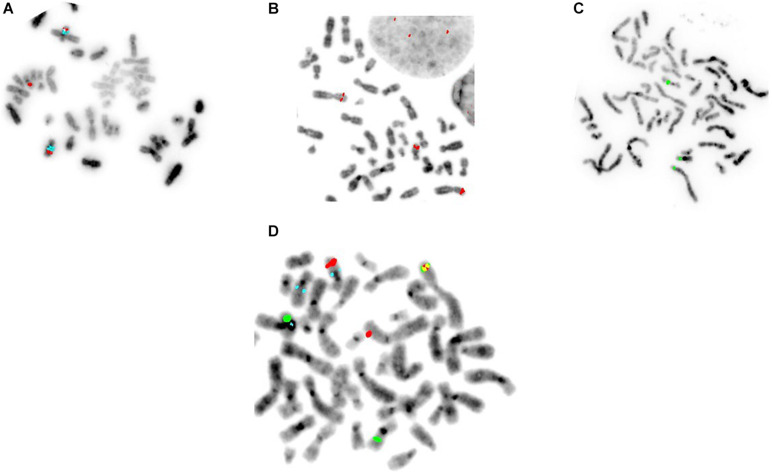
Specific FISH-EA probes for the translocation event(s) seen in the BEC model. Multiple probes for the same loci were tested to find the ideal probe spanning the breakpoints. **(A**–**C)** The ideal probes for Chr2 and Chr16, respectively. **(D)** Combining two FISH-EA probes specific for breakpoints on Chr2p22 (red) and Chr16q22 (green), respectively, the fusion could be visualized as a yellow signal resulting from the fusion of the two partial segments of the probes complementary to the respective chromosome sequences. A segment of Chr2 (red) is translocated to Chr10 (marked by aqua centromere probe).

The BAC-FISH probes hybridize to homologous chromosomes and emit two strong signals of the same color. A third signal of the same color appears in the event of a translocation, due to breakage of the probe that spans the breakpoints, as seen in the BEC40W cells (Figures 2A–C). The BAC-FISH probes (red and green) from breakpoints on two different chromosomes appear as a fusion signal (yellow), when paired together in a FISH-EA assay (Figure 2D). The fusion signals observed in the replicates of BEC40W cells were specific and sensitive to the translocations and not found in the naïve BAR-T or BEC20W or the control BAR-T cells growing without exposure to ABS for up to 60 weeks.

### The Translocations Occur in Human EAC and Not in Normal Esophagus or Non-dysplastic BE

**FIGURE 3:**
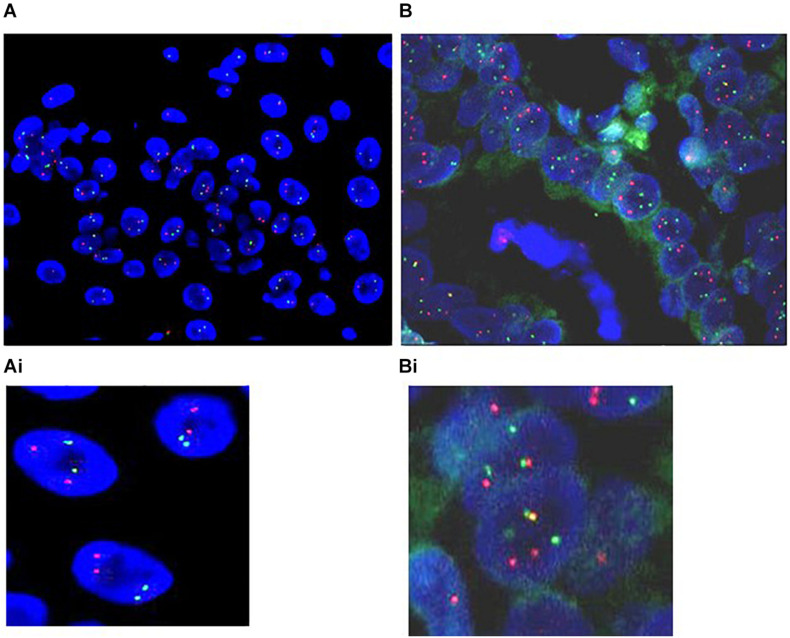
Translocations occur in human EAC and not in paired normal esophageal epithelium and non-dysplastic BE. Representative pictures of **(A)**. The non-dysplastic BE tissue: two signals in red and green representing two (Chr2 and Chr16) normal homologous chromosome pairs. **(Ai)** Enlargement of the normal signal. **(B)** FISH signal from paraffin-embedded EAC tumor tissue using BAC-FISH probes for Chr2p22 (red) and Chr16q22 (green) breakpoints, showing a fusion (yellow). The red and green signals denote intact parent chromosomes, and the yellow signal marks the fusion of the two chromosomes. Some cells also have three or more signals of each color representing breakage of chromosomes but no fusion. **(Bi)** Enlarged view of the FISH signals in tumor tissue.

Four of the 10 EAC tissues screened showed multiple cells with 3 red and green signals and some with a fusion signal (yellow) when we used BAC-FISH clones for chromosomes 2 (red) and 16 (green) (Figures 3B,Bi). However, we observed no fusions in the tissues when we combined BAC-FISH probes for Chr10 with probes for Chr2 or Chr16. All three pairs of normal squamous epithelium (results not shown) and non-dysplastic BE tissue samples tested were negative for fusions and showed two signals of each color on chromosomes 2, 10, and 16. Normal signal from probe set 1 (2p22;16q22) in non-dysplastic BE tissue shown in (Figures 3A,Ai).

### The Chromosomal Translocations Alter the Transcriptional Landscape of the Barrett’s Epithelial Cells

**FIGURE 4:**
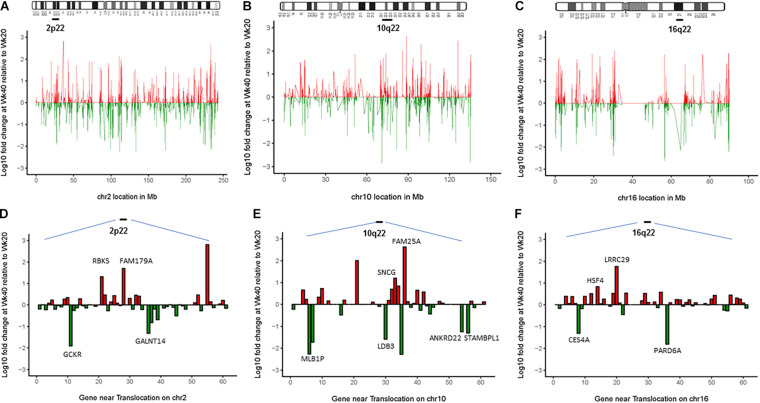
The chromosomal translocations alter the transcriptional landscape of the Barrett’s epithelial cells. Top panel **(A–C)** represents the entire altered transcriptional landscape of the three chromosomes 2, 10, and 16 in BEC40W compared with BEC20W cells (red: upregulation, green: downregulation of transcript levels). Bottom panel **(D–F)** shows log10 fold changes in FPKM transcript levels of genes in the chromosome loci spanned by FISH-EA probes (2p22, 10q22, and 16q22 marked by a bar). The graph represents fold changes in FPKM transcript levels of known genes located in the same regions. Genes altered more than twofold (*p* < 0.025) are listed in the tables with individual fold changes shown in red/green representing up/downregulation. The complete list of genes in these regions and the fold change in their transcript levels between BEC40W and BEC20W cells is provided in Tables 2–4. Random genes were selected, and expression levels were confirmed by qPCR (genes with LOC entries are pictured but not labeled on the figure).

Since chromosomal translocations are known to alter the transcription of genes at or near the breakpoint regions, the transcriptional profile (RNAseq) of the whole chromosomes 2, 10, and 16, as well as breakpoint regions were compared between the BEC20W cells (without translocations) and the BEC40W cells (with translocations). Genes on either side of the center of the chromosome segments spanned by the FISH-EA probes (2p22, 10q22, and 16q22) (Figure 4) altered more than twofold in transcript levels between BEC40W vs. BEC20W are listed in Tables 2–4 respectively. On 2p22: upregulated: FNDC4 (2.2-fold), RBKS (20.6-fold), FOSL2 (3.0-fold), SPDYA (2.7-fold), FAM179A (50.6-fold), CLIP4 (2-fold), YPEL5 (2.9-fold), and LBH (2.6-fold), RASGRP3 (2.9-fold); downregulated: GCKR (81.4-fold), GALNT14 (20.6-fold), CAPN14 (6.6-fold), XDH (4.8-fold), and NLRC4 (3.2-fold). On 10q22: upregulated: MMRN2 (4.9-fold), SNCG (15.9-fold), AGAP11 (6.9-fold), FAM25A (430.4-fold), FAM22D (3.7-fold); downregulated: MBL1P (52.5-fold), LDB3 (39.2-fold), PAPSS2 (2.0-fold), ANKRD22 (17.9-fold), and STAMBPL1 (20-fold). On 16q22: upregulated: PRAD (2.5-fold), CES2 (2.4-fold), B3GNT9 (2.5-fold), TRADD (3.2-fold), HSF4 (6.7-fold), ELMO3 (3.3-fold), LRRC29 (56.6-fold), SLC9A5 (3.4-fold), HSD11B2 (3.4-fold), ACD (3.9-fold), SLC12A4 (2.7-fold), ESRP2 (2.8-fold), and PLA2G15 (2.1-fold); downregulated: CES4A (20.3-fold), FHOD1 (2.8-fold), and PARD6A (64.3-fold). Lists of all the genes on 2p22, 10q22, and 16q22 and fold change in their transcript levels are provided as Supplementary Tables 2–4.

**TABLE 2:** List of known genes on 2p22 locus with more than twofold change in transcript levels between BEC20W and BEC40W cells, as represented in Figure 4.

**Gene name**	**Gene location (Hg19)**		**Fold change in FPKM transcript levels (BEC40W/BEC20W)**
	**Start**	**End**	
FNDC4	27714749	27718126	2.2
GCKR	27719705	27746550	–81.4
RBKS	28004265	28561767	20.6
FOSL2	28615778	28637516	2.9
SPDYA	29033699	29093175	2.7
FAM179A	29204163	29275096	50.6
CLIP4	29338307	29406679	2.0
YPEL5	30369749	30383399	2.9
LBH	30454396	30482899	2.6
GALNT14	31133332	31361571	–20.6
CAPN14	31395921	31440411	–6.6
XDH	31557187	31637611	–4.8
NLRC4	32449517	32490812	–3.2
RASGRP3	33661415	33789798	2.9

*Negative values indicate downregulation at BEC40W compared with BEC20W.*

**TABLE 3:** List of known genes on 10q22 locus with more than twofold change in transcript levels between BEC20W and BEC40W cells, as represented in Figure 4.

**Gene name**	**Gene location (Hg19)**		**Fold change in transcript levels**
MBL1P	81664653	81691557	–52.5
LDB3	88428205	88495824	–39.2
MMRN2	88695297	88717425	4.9
SNCG	88718287	88723017	15.9
AGAP11	88728187	88769960	6.9
FAM25A	88780045	88784487	430.4
FAM22D	89117476	89130452	3.7
PAPSS2	89419475	89507462	2.7
ANKRD22	90562486	90611732	17.9
STAMBPL1	90640025	90683244	20.0

*Negative values indicate down-regulation at BEC40W compared with BEC20W.*

**TABLE 4:** List of known genes on 16q22 locus with more than twofold change in transcript levels between BEC20W and BEC40W cells, as represented in Figure 4.

**Gene name**	**Gene location**		**Fold change in transcript levels**
	**Start**	**End**	
RRAD	66955581	66959439	2.5
CES2	66968346	66978994	2.4
CES4A	67022491	67043659	–20.3
B3GNT9	67143914	67184902	2.5
TRADD	67188088	67193812	3.2
HSF4	67193890	67203848	6.7
ELMO3	67233027	67237927	3.3
LRRC29	67241041	67260901	56.6
FHOD1	67263291	67281425	–2.8
SLC9A5	67282854	67306094	3.4
HSD11B2	67465035	67471454	3.4
ACD	67679029	67694718	3.9
PARD6A	67694850	67696681	–64.3
SLC12A4	67973786	68002597	2.7
ESRP2	68119268	68270136	2.8

*Negative values indicate down-regulation at BEC40W compared with BEC20W.*

## Discussion

Chromosome translocations are unique “biomarkers” in many malignancies and may present targets for therapeutic interventions. However, translocations are mostly described in hematological cancers, role of such events is reported in more than 20% of all cancer associated morbidity (Mitelman et al., 2007). It is therefore significant that we found recurrent translocations in samples from human esophageal adenocarcinoma tissues tested in this study. The only other study that reports fusions in EA is a computational analysis of 170 RNAseq datasets from patients with EAC. They identified fusions in a subset of 3.33–11.67% individuals (Blum et al., 2016). Five esophageal adenocarcinoma cell lines (FLO-1, SKGT4, OE33, OE19, and EsoAd) were tested and found to be negative for those fusions (Blum et al., 2016). We used the same bioinformatics methods as Blum et.al. on the RNAseq datasets of BEC model and the same EAC cell lines to screen for any fusions relevant to the t(2;10;16), but could not succeed. This could be due to the unique karyotype and genomic signature of the EA cell lines (Contino et al., 2016). The absence of fusion transcripts in our datasets could be either because the structural changes involve “gene deserts,” such as those described in band 10q22 (Ovcharenko et al., 2005; Chen et al., 2009) or due to involvement of non-coding RNAs or non-transcribed regions of the genome such as the promoter regions. The fusions may also have gone undetected simply due to limitations in the software algorithms (Kumar et al., 2016).

The chromosomal loci involved in the translocation events described in this report are significant in BE and EAC literature. Therefore, failure to detect fusion transcripts from the translocations does not exclude the possibility of alternative genomic changes resulting from these chromosomal translocations. Loss of 10q21.3 and 16q23.1 in the known fragile sites FRA10D and FRA16D were previously reported in BE (Gu et al., 2010). Loss of WWOX gene on the 16q23.1 locus is associated with 47.4% of LGD in BE (Gu et al., 2010), and the FOXF1 gene at 16q24 is associated with increased genetic risk for susceptibility to BE (Su et al., 2012). Loss of PAPSS2 at 10q22 is associated with poor prognosis in patients with resected Barrett’s adenocarcinoma and other gastroesophageal junction cancers (Peters et al., 2010). The chromosomal segments involved in the translocations in this study also harbor other genes known to participate in carcinogenesis and metastasis. Upregulation of FOSL2 at 2p22 is associated with colon cancer (Wang et al., 2014), and increased levels of FNDC4 transcripts are associated with increased inflammation in mouse models and in IBD patients (Bosma et al., 2016). Upregulation of YPEL5 is associated with action of erlotinib in non-small cell lung cancer (Wu, 2018). Highly invasive, ER-negative basal human breast cancers show overexpression of the LBH gene (Rieger et al., 2010). The occurrence of FAM179A-ALK fusion has been reported in non-small cell lung cancer (Yan et al., 2020). CAPN14 gene is highly expressed in the esophagus and is associated with epithelial barrier impairment observed in esosinophilic esophagitis (Davis et al., 2016). In the NLRC4 inflammasome, upregulation is a marker of poor prognosis in gliomas although the role of inflammasomes in tumors continues to evolve (Lim et al., 2019). The RASGRP3, a RAS activator is known to play a role in several cancers, including breast (Nagy et al., 2014), papillary thyroid (Qiu et al., 2017), and prostate cancers (Yang et al., 2010). The MMRN2 gene located on 10q22 interacts with the VEGF pathway to promote angiogenesis and hence tumor growth (Lorenzon et al., 2012). The ANKRD22 is reported to promote non-small cell lung cancer (Yin et al., 2017), and STAMBPL1 is oncogenic in gastric cancer (Yu et al., 2019). Loss of epigenetic control of SNCG gene is a marker of metastasis in several cancers (Jin et al., 2012). PAPSS2 is also commonly lost along with PTEN in prostate cancer xenografts (Hermans et al., 2004). Some of the known oncogenic genes on 16q22 are RRAD, a member of the Ras family, known to be associated with several cancers including breast cancer, leukemia, lymphoma, and glioma (Kim et al., 2019). Reduced transcript levels of the CES2 gene have been reported in colorectal cancer (Ishimine et al., 2018). ELMO3, in coordination with CDX2 plays a role in cellular migration in the intestine (Coskun et al., 2010) and during metastasis in lung cancer (Soes et al., 2014). The HSF4 gene, involved in cellular senescence, suppresses evolution of spontaneous tumors arising in *p53*- or *Arf*-deficient mice (Jin et al., 2012). The actin-associated formin homology 2 domain containing protein 1 (FHOD1) gene is upregulated in melanomas and modifies proliferation and tumor growth (Peippo et al., 2017), and the HSD11B2 plays a role in metastasis in colorectal cancer (Chen et al., 2020).

The BEC model enabled us to customize the BAC-FISH probes in metaphase cells where we can visualize the breakpoints after comparing the BEC0 and BEC20W cells with the BEC30W and later cells. However, we did not find fusion signals involving Chr10, perhaps because primary human BE and EAC tissues are complex, and application of the FISH-EA from cell culture to primary tissues presents difficulties due to presence of secondary chromosomal structures variation in chromosome numbers and juxtaposition of cell layers on 4 μm tissue sections. The FISH-EA assay needs more optimization with sensitive and repeat free DNA probes. Using esophageal mucosal brushings may eliminate the technical difficulty of signal visualization due to overlap of cell layers (Geisinger, 1995; Brankley et al., 2006; Timmer et al., 2014). Testing a larger set of stage-specific primary tissues will determine if any of the translocations is a predictor of progression in BE patients. The BAR-T and the BEC cell lines recapitulate molecular signaling events in gastroesophageal reflux-induced Barrett’s pathogenesis, as reported earlier (Jaiswal et al., 2007; Zhang et al., 2007, 2009, 2019; Bajpai et al., 2008, 2012, 2013). Therefore, it may be noted that the recurrent chromosome events in question occur in the BEC30W cells prior to development of malignant characteristics in BEC60W cells and may signify precursor events in BE carcinogenesis. Similar somatic chromosomal aberrations have also been implicated in the evolution of BE to EA by other investigators (Maley et al., 2006; Martinez et al., 2016).

In summary, the FISH-EA assay detects chromosome translocations that are specific to malignantly transformed BE cells and EAC tissues. The transcriptional alterations identified near the breakpoints of chromosomes 2, 10, and 16 include genes known to promote carcinogenesis in several cancers, including EA. The possibility of combining the FISH-EA probes with histology or comparison of the performance of the FISH-EA assay with existing esophageal cancer detection assays merits further investigation. This assay adds a new tool to the ongoing quest for reliable biomarkers for surveillance and patient risk stratification in BE carcinogenesis. The ease of the FISH-EA assay and the uniqueness of chromosomal translocations make this method more specific, sensitive, and easy to adapt in all cytogenetic laboratories.

## Data Availability Statement

The original contributions presented in the study are included in Supplementary Material.

## Author Contributions

MB led the conception and design, analysis, interpretation and compilation of data, and writing the manuscript. AP performed the interpretation of gene expression data and all computational analyses. KD, AC, and PJ helped with tissue acquisition. PJ confirmed histology evaluations on BE and EAC tissue sections stained with H&E. KB performed the FISH and subsequent data acquisition. HA participated in design of studies, supervised KB, and performed the interpretation of images and data. MB and HA revised the manuscript critically for important intellectual content. All authors approved the final version of the manuscript to be published.

## Conflict of Interest

MB, HA, and KD have a patent application U.S. Application No. 16/083,055 pending for the FISH-EA probes. The remaining authors declare that the research was conducted in the absence of any commercial or financial relationships that could be construed as a potential conflict of interest. The handling editor declared a past co-authorship with one of the authors AP.

## Supplementary Material

Supplementary Figures 1–3 H&E sections of the normal, non-dysplastic BE, and esophageal adenocarcinoma tissues presented in Figure 3 for this study.
