# Supplementary figures and images for: Recurring Translocations in Barrett’s Esophageal Adenocarcinoma
Source: Front Genet. 2021 Jun 9;12:674741. doi: 10.3389/fgene.2021.674741 (PMC8220202; doi:10.3389/fgene.2021.674741)

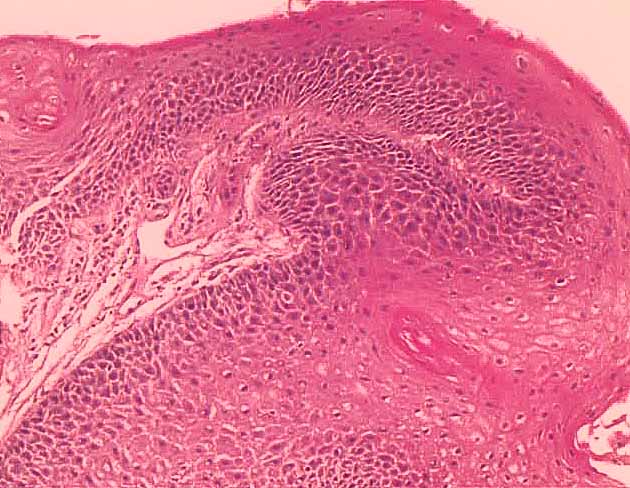

Supplement: Supplementary Figures 1–3 — H&E sections of the normal, non-dysplastic BE, and esophageal adenocarcinoma tissues presented in Figure 3 for this study. [file Image_1.JPEG]

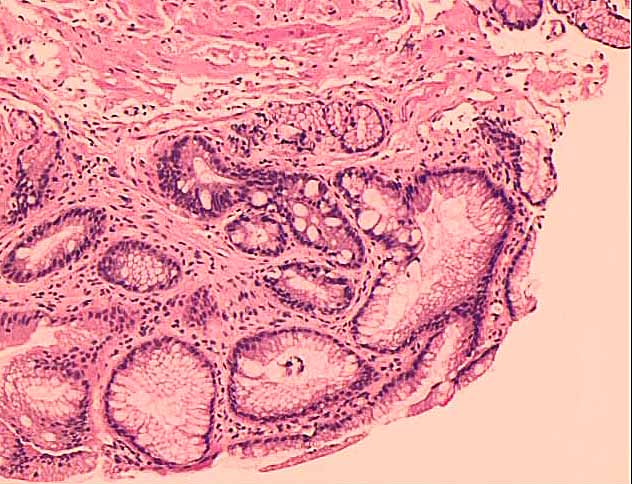

Supplement: Supplementary file 2 [file Image_2.JPEG]

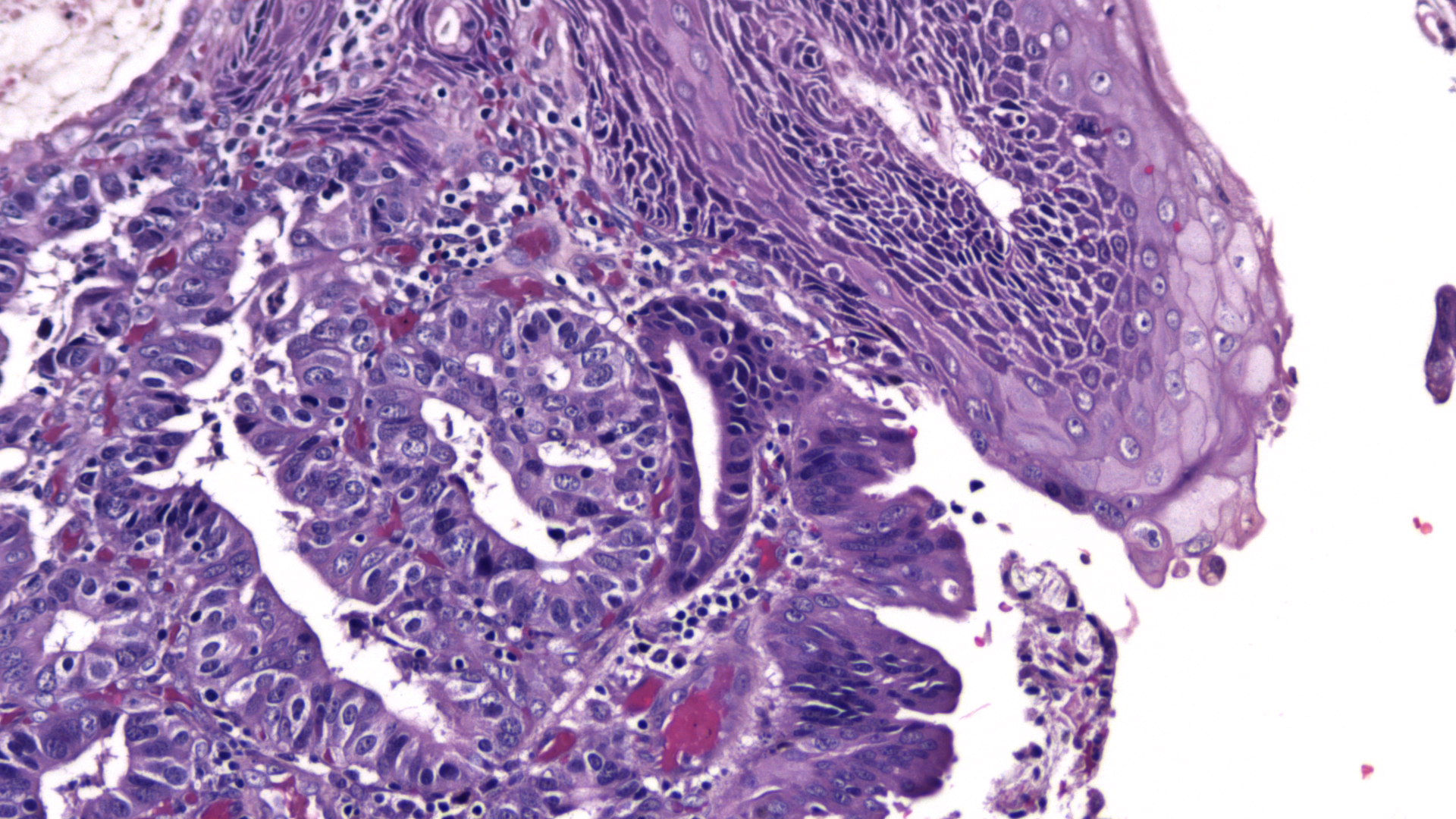

Supplement: Supplementary file 3 [file Image_3.PNG]
