# Supplementary material for: Recurring Translocations in Barrett’s Esophageal Adenocarcinoma
Source: Front Genet. 2021 Jun 9;12:674741. doi: 10.3389/fgene.2021.674741 (PMC8220202; doi:10.3389/fgene.2021.674741)
Supplement: Supplementary file 5 [file Table_2.DOCX]

Table 2: List of genes on 2p22 locus with change in transcript levels between BEC20 and BEC40W cells as represented in Fig: 4

| **Gene name** | **Gene Location (Hg19)** | | **Fold change in transcripts between BEC40W/BEC20W** |
| --- | --- | --- | --- |
|  | **start** | **end** |  |
| GTF3C2 | 27548720 | 27579868 | 0.6 |
| EIF2B4 | 27587218 | 27593324 | 1.0 |
| SNX17 | 27593388 | 27599994 | 0.6 |
| ZNF513 | 27600101 | 27603593 | 1.2 |
| FTH1P3 | 27604060 | 27632496 | 1.7 |
| PPM1G | 27604060 | 27632496 | 0.6 |
| NRBP1 | 27651472 | 27665124 | 0.9 |
| IFT172 | 27665232 | 27712571 | 0.8 |
| KRTCAP3 | 27665232 | 27712571 | 1.9 |
| FNDC4 | 27714749 | 27718126 | 2.2 |
| GCKR | 27719705 | 27746550 | 0.0 |
| C2orf16 | 27799388 | 27805589 | 0.6 |
| ZNF512 | 27805892 | 27845963 | 0.7 |
| CCDC121 | 27848505 | 27917847 | 1.9 |
| GPN1 | 27848505 | 27917847 | 0.9 |
| SLC4A1AP | 27848505 | 27917847 | 0.7 |
| SUPT7L | 27848505 | 27917847 | 1.0 |
| MRPL33 | 27994583 | 28002608 | 1.0 |
| BRE | 28004265 | 28561767 | 0.9 |
| LOC100302650 | 28004265 | 28561767 | 1.0 |
| RBKS | 28004265 | 28561767 | 20.6 |
| FOSL2 | 28615778 | 28637516 | 2.9 |
| PLB1 | 28718937 | 28866653 | 0.5 |
| PPP1CB | 28974613 | 29025806 | 1.4 |
| SPDYA | 29033699 | 29093175 | 2.7 |
| TRMT61B | 29033699 | 29093175 | 1.2 |
| WDR43 | 29117532 | 29171080 | 0.9 |
| FAM179A | 29204163 | 29275096 | 50.6 |
| C2orf71 | 29284557 | 29297127 | 1.0 |
| CLIP4 | 29338307 | 29406679 | 2.0 |
| ALK | 29415639 | 30144432 | 0.6 |
| YPEL5 | 30369749 | 30383399 | 2.9 |
| LBH | 30454396 | 30482899 | 2.6 |
| LCLAT1 | 30670122 | 30867091 | 0.6 |
| CAPN13 | 30945637 | 31030311 | 1.0 |
| GALNT14 | 31133332 | 31361571 | 0.0 |
| CAPN14 | 31395921 | 31440411 | 0.2 |
| EHD3 | 31456879 | 31491260 | 0.7 |
| XDH | 31557187 | 31637611 | 0.2 |
| SRD5A2 | 31749655 | 31806040 | 1.0 |
| MEMO1 | 32092893 | 32235698 | 0.7 |
| DPY30 | 32248971 | 32264844 | 0.9 |
| SPAST | 32288679 | 32382706 | 1.0 |
| SLC30A6 | 32390909 | 32449181 | 0.8 |
| NLRC4 | 32449517 | 32490812 | 0.3 |
| YIPF4 | 32502957 | 32531658 | 1.0 |
| BIRC6 | 32582095 | 32843965 | 0.9 |
| TTC27 | 32853086 | 33046118 | 0.6 |
| LOC100271832 | 33050509 | 33171202 | 1.0 |
| LOC285045 | 33050509 | 33171202 | 1.0 |
| LTBP1 | 33172368 | 33624575 | 1.3 |
| RASGRP3 | 33661415 | 33789798 | 2.9 |
| FAM98A | 33808728 | 33824362 | 0.5 |
| MYADML | 33951127 | 33953284 | 1.0 |
| LOC100288911 | 36581891 | 36582713 | 647.9 |
| CRIM1 | 36583369 | 36778278 | 1.4 |
| FEZ2 | 36779403 | 36825332 | 0.8 |
| VIT | 36923832 | 37041937 | 1.0 |
| STRN | 37075471 | 37193615 | 1.1 |
| HEATR5B | 37208152 | 37311485 | 1.7 |
| CCDC75 | 37311593 | 37323738 | 0.7 |
