# Supplementary material for: Recurring Translocations in Barrett’s Esophageal Adenocarcinoma
Source: Front Genet. 2021 Jun 9;12:674741. doi: 10.3389/fgene.2021.674741 (PMC8220202; doi:10.3389/fgene.2021.674741)
Supplement: Supplementary file 6 [file Table_3.DOCX]

Table 3: List of genes on 10q22 locus with change in transcript levels between BEC20 and BEC40W cells as represented in Fig: 4

| **Gene name** | **Gene Location (Hg19)** | | **Fold change in transcripts between BEC40W/BEC20W** |
| --- | --- | --- | --- |
|  | **start** | **end** |  |
| EIF5AL1 | 81272356 | 81276192 | 0.6 |
| SFTPA2 | 81315607 | 81320163 | 1.0 |
| SFTPA1 | 81370694 | 81375199 | 1.0 |
| LOC650623 | 81442730 | 81448650 | 4.6 |
| LOC642361 | 81585657 | 81587358 | 1.7 |
| LOC100288974 | 81664653 | 81691557 | 0.0 |
| MBL1P | 81664653 | 81691557 | 0.0 |
| SFTPD | 81697495 | 81708861 | 1.0 |
| C10orf57 | 81805988 | 81852307 | 2.2 |
| LOC219347 | 81805988 | 81852307 | 5.3 |
| PLAC9 | 81892257 | 81904784 | 1.0 |
| ANXA11 | 81914879 | 81965328 | 1.5 |
| MAT1A | 82031575 | 82049434 | 1.0 |
| DYDC1 | 82095861 | 82116500 | 1.0 |
| DYDC2 | 82116557 | 82127829 | 1.0 |
| C10orf58 | 82168241 | 82192753 | 0.3 |
| TSPAN14 | 82214037 | 82282391 | 1.6 |
| SH2D4B | 82297657 | 82406316 | 1.0 |
| NRG3 | 83635069 | 84746935 | 1.0 |
| GHITM | 85899184 | 85913311 | 1.0 |
| C10orf99 | 85933553 | 85945050 | 101.5 |
| CDHR1 | 85954411 | 85979376 | 1.0 |
| LRIT2 | 85980248 | 85985284 | 1.0 |
| LRIT1 | 85991275 | 86001217 | 1.0 |
| RGR | 86004808 | 86018944 | 1.0 |
| FAM190B | 86088409 | 86278276 | 1.3 |
| GRID1 | 87359311 | 88126250 | 1.0 |
| WAPAL | 88195012 | 88281541 | 0.8 |
| OPN4 | 88414313 | 88426216 | 1.0 |
| LDB3 | 88428205 | 88495824 | 0.0 |
| BMPR1A | 88516395 | 88684945 | 1.5 |
| MMRN2 | 88695297 | 88717425 | 4.9 |
| SNCG | 88718287 | 88723017 | 15.9 |
| AGAP11 | 88728187 | 88769960 | 6.9 |
| C10orf116 | 88728187 | 88769960 | 0.0 |
| FAM25A | 88780045 | 88784487 | 430.4 |
| GLUD1 | 88809958 | 88854776 | 1.3 |
| FAM35A | 88854952 | 88951222 | 0.7 |
| FAM22A | 88985204 | 88994733 | 1.1 |
| LOC439994 | 88998423 | 89103331 | 4.5 |
| LOC728190 | 88998423 | 89103331 | 0.5 |
| FAM22D | 89117476 | 89130452 | 3.7 |
| MINPP1 | 89264222 | 89313218 | 0.9 |
| PAPSS2 | 89419475 | 89507462 | 0.4 |
| ATAD1 | 89512874 | 89577917 | 0.7 |
| CFLP1 | 89578069 | 89605369 | 1.0 |
| KILLIN | 89618917 | 89623194 | 1.4 |
| PTEN | 89623194 | 89728532 | 1.1 |
| RNLS | 90033620 | 90343082 | 1.0 |
| LIPJ | 90346518 | 90366733 | 1.0 |
| LIPF | 90424145 | 90438572 | 1.0 |
| LIPK | 90484300 | 90512513 | 1.0 |
| LIPN | 90521162 | 90537999 | 1.0 |
| ANKRD22 | 90562486 | 90611732 | 0.1 |
| LIPM | 90562486 | 90611732 | 1.0 |
| STAMBPL1 | 90640025 | 90683244 | 0.0 |
| ACTA2 | 90694830 | 90775542 | 1.4 |
| FAS | 90694830 | 90775542 | 0.7 |
| FAS-AS1 | 90694830 | 90775542 | 1.0 |
| CH25H | 90965693 | 90967071 | 1.0 |
| LIPA | 90973325 | 91011660 | 1.3 |
