# Supplementary material for: Recurring Translocations in Barrett’s Esophageal Adenocarcinoma
Source: Front Genet. 2021 Jun 9;12:674741. doi: 10.3389/fgene.2021.674741 (PMC8220202; doi:10.3389/fgene.2021.674741)
Supplement: Supplementary file 7 [file Table_4.DOCX]

Table 4: List of genes on 16q22 locus with change in transcript levels between BEC20 and BEC40W cells as represented in Fig: 4

| **Gene name** | **Gene Location (Hg19)** | | **Fold change in transcripts between BEC40W/BEC20W** |
| --- | --- | --- | --- |
|  | **start** | **end** |  |
| CA7 | 66878281 | 66888049 | 1.0 |
| PDP2 | 66914382 | 66925002 | 0.7 |
| CDH16 | 66942024 | 66952766 | 1.0 |
| RRAD | 66955581 | 66959439 | 2.5 |
| FAM96B | 66965957 | 66968320 | 1.1 |
| CES2 | 66968346 | 66978994 | 2.4 |
| CES3 | 66995131 | 67009052 | 1.1 |
| CES4A | 67022491 | 67043659 | 0.0 |
| CBFB | 67063049 | 67134958 | 0.7 |
| B3GNT9 | 67143914 | 67184902 | 2.5 |
| C16orf70 | 67143914 | 67184902 | 1.1 |
| TRADD | 67188088 | 67193812 | 3.2 |
| FBXL8 | 67193890 | 67203848 | 1.0 |
| HSF4 | 67193890 | 67203848 | 6.7 |
| KIAA0895L | 67204404 | 67217883 | 1.1 |
| NOL3 | 67204404 | 67217883 | 1.8 |
| EXOC3L | 67218281 | 67224107 | 1.0 |
| E2F4 | 67226067 | 67232821 | 0.9 |
| ELMO3 | 67233027 | 67237927 | 3.3 |
| LRRC29 | 67241041 | 67260901 | 56.6 |
| TMEM208 | 67261015 | 67263182 | 1.2 |
| FHOD1 | 67263291 | 67281425 | 0.4 |
| SLC9A5 | 67282854 | 67306094 | 3.4 |
| KCTD19 | 67311412 | 67360661 | 1.0 |
| PLEKHG4 | 67311412 | 67360661 | 1.0 |
| LRRC36 | 67360746 | 67419109 | 1.0 |
| TPPP3 | 67423711 | 67427421 | 1.0 |
| ZDHHC1 | 67428321 | 67450339 | 1.5 |
| HSD11B2 | 67465035 | 67471454 | 3.4 |
| ATP6V0D1 | 67471916 | 67515089 | 1.3 |
| AGRP | 67516473 | 67517716 | 1.0 |
| FAM65A | 67562716 | 67580691 | 1.3 |
| CTCF | 67596309 | 67673088 | 0.7 |
| ACD | 67679029 | 67694718 | 3.9 |
| RLTPR | 67679029 | 67694718 | 1.0 |
| PARD6A | 67694850 | 67696681 | 0.0 |
| C16orf48 | 67696849 | 67700628 | 1.2 |
| C16orf86 | 67700716 | 67702661 | 1.0 |
| GFOD2 | 67708435 | 67753273 | 1.8 |
| RANBP10 | 67757004 | 67840555 | 1.7 |
| TSNAXIP1 | 67841009 | 67861971 | 0.9 |
| CENPT | 67862059 | 67905219 | 1.3 |
| NUTF2 | 67862059 | 67905219 | 1.7 |
| THAP11 | 67862059 | 67905219 | 0.9 |
| EDC4 | 67906998 | 67918406 | 1.2 |
| NRN1L | 67918780 | 67920271 | 1.0 |
| CTRL | 67927174 | 67965778 | 1.0 |
| PSKH1 | 67927174 | 67965778 | 1.1 |
| PSMB10 | 67968406 | 67970753 | 1.0 |
| LCAT | 67973786 | 68002597 | 1.8 |
| SLC12A4 | 67973786 | 68002597 | 2.7 |
| DPEP3 | 68009565 | 68014452 | 1.0 |
| DPEP2 | 68021292 | 68033364 | 1.0 |
| DDX28 | 68055176 | 68113184 | 0.6 |
| DUS2L | 68055176 | 68113184 | 0.5 |
| ESRP2 | 68119268 | 68270136 | 2.8 |
| NFATC3 | 68119268 | 68270136 | 1.1 |
| PLA2G15 | 68279246 | 68294961 | 2.1 |
| SLC7A6 | 68298422 | 68344868 | 1.8 |
| SLC7A6OS | 68298422 | 68344868 | 1.2 |
| PRMT7 | 68344876 | 68391169 | 0.7 |
